# Contamination of sea urchin *Mesocentrotus nudus* by radiocesium released during the Fukushima Daiichi Nuclear Power Plant accident

**DOI:** 10.1371/journal.pone.0269947

**Published:** 2022-08-15

**Authors:** Mst. Nazira Akhter Rithu, Akira Matsumoto, Naoto Hirakawa, Yukari Ito, Hisayuki Arakawa

**Affiliations:** 1 Tokyo University of Marine Science and Technology, Minato, Tokyo, Japan; 2 Soma Branch, Fishery Office of the Fukushima Prefectural Government, Fukushima, Japan; 3 Fukushima Prefectural Research Institute of Fisheries Resources, Fukushima, Japan; University of South Carolina, UNITED STATES

## Abstract

Countless marine organisms were polluted with radioactive materials that were dispersed when the Fukushima Daiichi Nuclear Power Plant (FDNPP) was damaged in 2011 by the Great East Japan Earthquake. The aim of this study was to determine the degree to which marine herbivorous sea urchins, *Mesocentrotus nudus*, were contaminated with radiocesium because of the accident. We collected samples of sea urchins from four locations in Fukushima prefecture (at the coast and offshore from the Yotsukura and Ena stations) and investigated how the ^137^Cs activity concentrations changed. The biological half-life (*T*_bio_) of ^137^Cs in the individual sea urchins was between 121 and 157 days. The ecological half-life (*T*_eco_) of ^137^Cs was 181–423 days and was high in places close to the FDNPP. The *T*_eco_ values in the sea urchins were longer than previously reported. The results infer that the food sources of the sea urchins around the Fukushima coast strongly influenced their uptake of ^137^Cs.

## Introduction

Massive quantities of radioactive materials leaked into the atmosphere after the Fukushima Daiichi Nuclear Power Plant (FDNPP) accident in Fukushima Prefecture, Japan, operated by the Tokyo Electric Power Company (TEPCO), sustained severe damage because of the Great East Japan Earthquake (Mw 9.0) and large tsunami occurred on 11 March 2011. About 80% (3.5–27 PBq) of the radioactive cesium (Cs) released into the atmosphere settled in the ocean [1, 2]. Radioactive Cs amounting to 3.5 PBq was transported directly out into the ocean [1]. The water mass with high levels of radioactive cesium flowed south from the FDNPP [1, 3–5] and contaminated many marine biota and seabed sediments along the southern coast and offshore area of Fukushima Prefecture [6–12]. Numerous researchers have observed temporal change in the radiocesium contamination of various marine organisms, including fish [13–15], invertebrates [16, 17], and algae [9, 18]. From monitoring data, these researchers showed that the concentrations tended to decrease over time, and that the radioactive concentrations in marine invertebrates decreased more quickly, such that the concentrations in many species were below the level of detection within three years of the FDNPP accident.

The variations in radioactive Cs concentrations in seawater, food, excreted material, and seafloor sediment (including reefs) need to be considered when analyzing the contamination of benthic invertebrates. In addition, researchers have examined how ingested contaminated sediments affected the digestive tract [7, 14, 17]. Elsewhere, researchers have reported that the uptake of radioactive Cs in benthic invertebrates depends on species-specific feeding habits, even when the surrounding sediments are highly contaminated [19]. Given the range of topics and findings so far, further research that includes food and the sediment environment is needed to clarify the mechanisms by which benthic organisms are contaminated by radioactive cesium.

*Mesocentrotus nudus*, a sea urchin, was an important fishery product in the Fukushima coastal area before the FDNPP accident. Monitoring data showed that the ^137^Cs activity concentration in *M*. *nudus* reached about 1000 Bq/kg-WW after the accident, then decreased gradually until it was below the detection limit (5.4–8.5 Bq/kg-WW) in 2015 [14, 20]. However, some studies reported that the decreasing trend in radiocesium concentrations in Echinoidea was not universal, and that the radiocesium concentrations in some species of this class, such as sea urchins, were relatively high [16, 17].

Echinoderms are primary consumers in ecosystems and may ingest food that is contaminated by radioactivity. We know that *M*. *nudus* mainly feed on large brown algae, but may be omnivorous, depending on the season [21]. It would be useful to have information about how the levels of radioactive materials in living organisms and their discharges changed in the period after the accident therefore, to understand whether radioactive materials have been transferred from food to primary consumers since the FDNPP accident.

The purpose of this study was to determine how the ^137^Cs contamination of the primary consumer sea urchin *M*. *nudus* from the FDNPP accident changed over time. To achieve this, the biological half-life (*T*_bio_) and ecological half-life (*T*_eco_) of radioactive cesium in *M*. *nudus* were investigated over the period from 426–2726 days after the accident. We also examined whether *M*. *nudus* consumed food with high ^137^Cs activity concentrations. Through these investigations, we examined how radioactive Cs was transferred to primary consumers after the FDNPP accident.

## Materials and methods

### Sample collection and rearing the sea urchin

We estimated the biological half-life (*T*_bio_) of ^137^Cs in sea urchins reared in laboratory conditions. Live *M*. *nudus* samples were collected from the Ena rocky reef fishing grounds (36.9413°N, 140.9481°E) in the northern part of Iwaki City, Fukushima. Once collected, the samples were immediately transported to the laboratory at the Fukushima Prefectural Fisheries Experimental Station (Shimokajiro, Iwaki City, Fukushima). At the laboratory, the sea urchins were cultured in an aquarium for 7 days to ensure the contents of the digestive tract were excreted before starting the experiment. The process to rear 7 sea urchin individuals was started on 31 May 2013. The rearing period was from May 2013 to August 2013, and 5 sea urchin samples were successfully reared in laboratory and survived for between 77 and 91 days. The 200-L rectangular rearing tank was divided into 6 sections, and one sea urchin was placed in each section. Each section measured 255 × 203 × 382 mm, and was filled to a depth of 300 mm with seawater that was pumped from the coast of Iwaki City. Kaeriyama (2017) reported the ^137^Cs activity concentrations of seawater at Onahama Port, Iwaki City and reported that the average concentration of ^137^Cs in seawater was 0.023±0.007 Bq /L during the sea urchin rearing period (S1 Fig) [22]. The seawater was filtered through sand and then poured into the tanks at a rate of 20–30 L/min. The sea urchins were fed until satiety with dried blades of kelp *Laminaria* spp., brought from Hokkaido, Northern Japan. No ^137^Cs was detected in the kelp.

The live sea urchin individuals were picked out from the rearing tank every week. On removal, the sea urchins were dried to remove any water, left in the air for 1 hour to drain as much of the internal water as possible, and then weighed. After measuring the body weight, each sea urchin was transferred into a 500 mL sealed container with a 90-mm inner diameter to measure the ^137^Cs counts. The shell length of the sea urchins on the first day of the experiment was 55.4±2.5 mm (S1 Table), and the individuals were almost the same size. The body weights increased and decreased during the experiment, but the shell length did not change significantly. One individual sea urchin was placed in a specimen bottle, so that the center of the bottle can be positioned in the center of the detector for the measurements. The ^137^Cs counts in the sea urchins was determined using gamma spectrometry in a closed-end coaxial high-purity germanium (HPGe) semiconductor detector (GEM20P4-70; Ortec, Tennessee, USA) at the Fukushima Prefectural Fisheries Experimental Station. The counting efficiency calibration of the HPGe detector was checked using volume standard sources (MX033U8PP for a 500 mL cylindrical container, Japan Radioisotope Assoc., Tokyo, Japan). The gamma rays from ^137^Cs were analyzed from the respective peaks in the energy spectrum for ^137^Cs (662 keV). Each sample weighed 0.063–0.086 kg and the measurement time for each sample was set at 5000 s. The radioactivity of a live sea urchin was measured weekly and the ^137^Cs counts represented the ^137^Cs activity [23].

The *T*_bio_ in days for each individual sea urchin was estimated from the total number of ^137^Cs counts in the ^137^Cs energy region (662 keV). We started to rear the sea urchins on 31 May 2013 (*t* = 0). The initial ^137^Cs counts (*C*_0_) and depuration rate constant (*λ*) were calculated from the exponential functional model in Eq (1) and the effective half-life (*T*_eff_) of ^137^Cs in the sea urchins was calculated from Eq (2). The *T*_bio_ of ^137^Cs in the live sea urchins was calculated from the *T*_eff_ and the physical half-life (*T*_p_) of ^137^Cs with Eq (3).

$$C_{t}=C_{0}e^{\left(‐\lambda t\right)},$$ (1)

$$T_{eff}=ln\;2/\lambda ,$$ (2)

$$1/T_{bio}=1/T_{eff}‐1/T_{p},$$ (3)

where *t* denotes the number of elapsed days, *C*_t_ represents the ^137^Cs counts at elapsed day *t*, and *C*_0_ represents the initial ^137^Cs counts. *λ* is the depuration rate constant that allows the effective half-life (*T*_eff_) to be calculated (2). The *T*_bio_ was calculated from the *T*_eff_ and the *T*_p_ (3). The *T*_p_ of ^137^Cs is 11013 days.

### Radiocesium concentrations of wild samples

**Fig 1:**
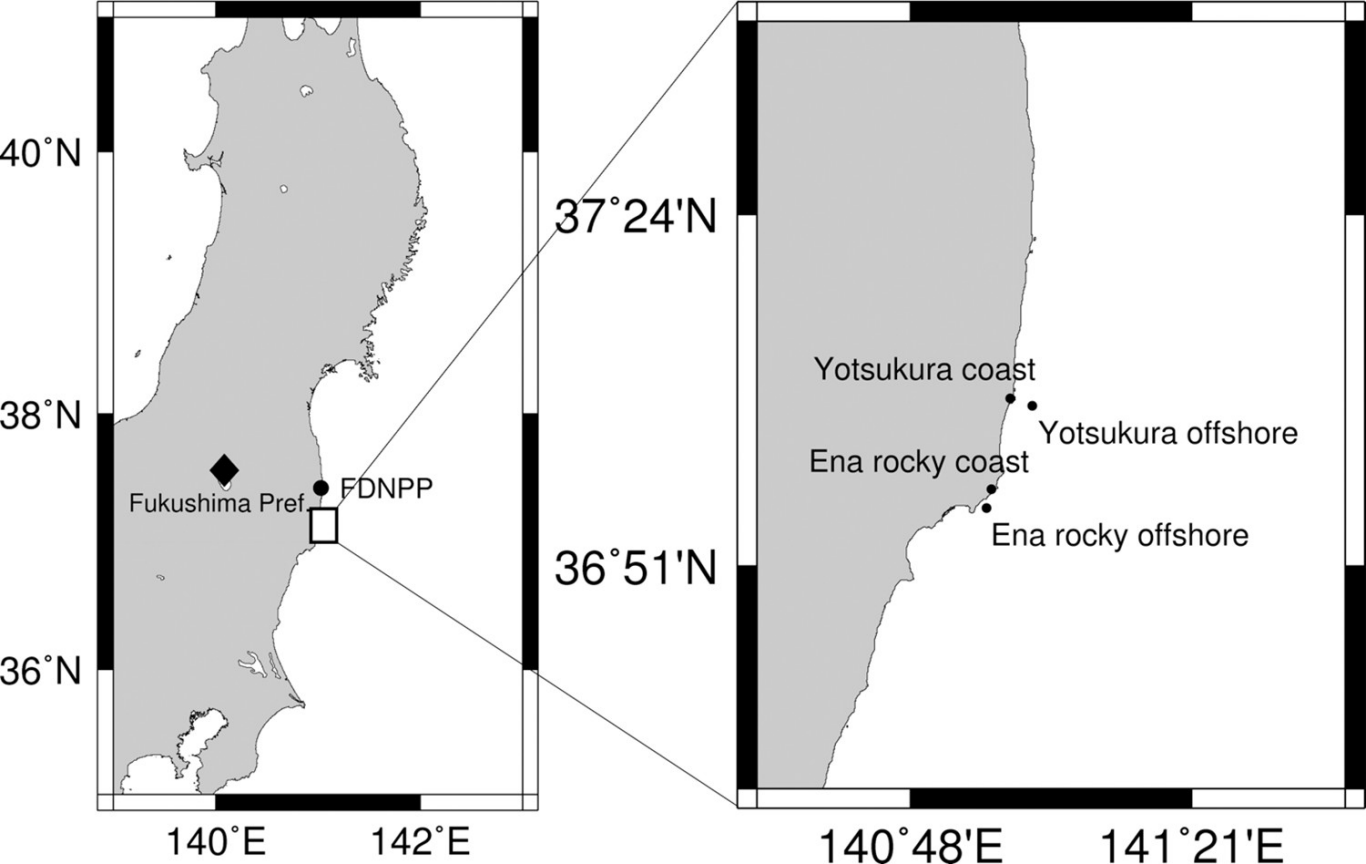
Sea urchin sampling sites on the Fukushima coast. The Yotsukura and Ena stations are about 35 and 50 km south of the Fukushima Daiichi Nuclear Power Plant (FDNPP), respectively.

To measure the ecological half-life (*T*_eco_) of radiocesium (^137^Cs and ^134^Cs) in wild sea urchins (*M*. *nudus*), live samples were collected from 4 locations in Iwaki City, Fukushima Prefecture, that were between 35 and 50 km from the FDNPP. The samples were collected from different water depths at the coast and offshore, to permit comparison between shallow and deep areas. The sampling sites were Yotsukura coast (37.112°N, 140.995°E, 35 km south of the FDNPP, 1–2 m depth), Yotsukura offshore (37.101°N, 141.038°E, 25–35 m depth), Ena rocky coast (36.971°N, 140.958°E, 50 km south of the FDNPP, 6 m depth), and Ena rocky offshore (36.9413°N, 140.9481°E, 8–16 m depth) (Fig 1).

Sea urchin samples were collected on 12 occasions from 20 July 2012 until 29 May 2014 (426–1175 days after the FDNPP accident), in May, July, October, November, and December of 2012; in January, February, May, and October of 2013; and in January, February, and May of 2014. Further samples were collected from the Yotsukura coast on 27 August 2018. The details of the sampling program are provided in S2 Table. Consent and special permission to collect wild samples (including samples for laboratory experiments) were gained from the Iwaki City Fisheries Cooperative Association and Fukushima Prefecture, respectively.

Once collected, the sea urchin samples were immediately transported to the laboratory. The sea urchin samples were washed with seawater and many of them were dissected to extract the gonads. To examine the contamination pattern, the samples were divided into two categories, namely whole body and gonads. About 20 individual sea urchins/gonads were crushed and collected in one 100-mL plastic container (U-8, AS ONE, Inc., Osaka), and the weights of the individual samples were measured (23 whole body and 63 gonad samples). All the samples were kept at −18°C until the radioactivity was measured (N = 86).

The radiocesium (^137^Cs and ^134^Cs) concentrations in the whole body and gonad samples (86 samples) were measured using a germanium (Ge) semiconductor detector (GEM20-70, SEIKO EG&G CO. LTD, Tokyo; resolution of <2.0 keV/1.33 MeV). The detector efficiency was calibrated with volume radioactivity standard gamma sources (MX033U8PP, Japan Radioisotope Association, Tokyo, Japan). Gamma rays from ^137^Cs and ^134^Cs were analyzed from the relevant peaks in the energy spectrum for ^137^Cs (662 keV) and ^134^Cs (605 keV and 796 keV). The samples weighed from 0.022 to 0.167 kg and the measurement time was set to 7200 s per sample. The radiocesium concentrations in sea urchin whole body and gonad samples were decay-corrected to the sample collection date and were presented as Bq/kg-wet weight (WW) [23]. Sea urchin samples in which cesium was not detected (n = 4) were dried to remove the moisture and were further analyzed in a well-type coaxial germanium detector (EGPC 250-P21, Canberra France S.A.S, less than 2.1 KeV/1.33 MeV of resolution) for measurement times of 113, 960–229, 940 s per sample.

The *T*_eco_ values of radiocesium (^137^Cs and ^134^Cs) in the sea urchins were calculated from the concentrations measured by the Ge semiconductor detector. The FDNPP accident occurred on 11 March 2011 (*t* = 0). The initial radiocesium concentration (*C*_0_) and the decay rate constant (*λ*_eco_) were calculated from an exponential functional model (Eq 1). The *T*_eff_ and the *T*_eco_ were calculated using Eqs (2) and (4), respectively. Any data below the detection limit (ND) were excluded from the *T*_eco_ calculations. The detection limit of the concentration was defined from the counting statistics as the concentration of three times the standard deviation.

$$1/T_{eco}=1/T_{eff}‐1/T_{p},$$ (4)

where *t* denotes the number of days since the FDNPP accident; *C*_0_ and *C*_t_ are the initial radiocesium concentrations on 11 March 2011 (*t* = 0) and the concentrations after *t* days, respectively. *λ* denotes the decay rate constant. ^137^Cs and ^134^Cs had *T*_*p*_ values of 11013 and 752.63 days, respectively.

### Apparent concentration factor (ACF)

The apparent concentration factor (ACF) was determined from data collected by the NRA for the ^137^Cs activity concentrations in seawater (https://www.nsr.go.jp/english/index.html) from 2012–2014, and data from August 2018 during the sea urchin sampling periods. Sampling points of seawater measured by the NRA were Yotsukura station (T-12) and Ena station (T-20). Seawater samples were collected to a depth of about 3 m below the sea surface.

### Statistical analysis

A generalized linear model (GLM) was used to determine the effects of the specific growth rate (hereinafter; SGR, % day^−1^) and elapsed days from the first measurement (31 May 2013) on the reduction rate of the Cs counts (*D*, day^−1^) in sea urchins during the rearing period. The sample ID (A–E) and SGR of the sea urchins and elapsed days were used as explanatory variables, and *D* in the sea urchins was the response variable in the GLM analysis. A normal distribution was applied to the response variable (*D*) and the best model was selected with the lowest Akaike information criterion (AIC). Moreover, a null model was built using the response variable only. The effect of the explanatory variables was determined by a maximum likelihood test with the best model and the null model. The correlations between the radiocesium concentrations in the sea urchins and elapsed days, and the ACF and elapsed days from the FDNPP accident, were determined with the Spearman’s rank test. Analysis of variance (ANOVA) was performed to demonstrate the spatial variation in the ^137^Cs activity concentrations in wild sea urchins. Different trends in the ^137^Cs counts in the rearing experiment and the ^137^Cs activity concentrations of the samples collected from the Yotsukura and Ena sites were tested by analysis of covariance (ANCOVA). Since the *T*_bio_ was calculated from the ^137^Cs counts and the *T*_eco_ was calculated from the ^137^Cs activity concentrations, the relative value of the ANCOVA test was the tendency to decrease. All the statistical analyses were performed using Microsoft Office Excel 365 (Microsoft, USA) and JMP software (JMP 13, SAS Institute Inc. Cary, NC, USA).

## Results

### Biological half-life of ^137^Cs in sea urchins

**Fig 2:**
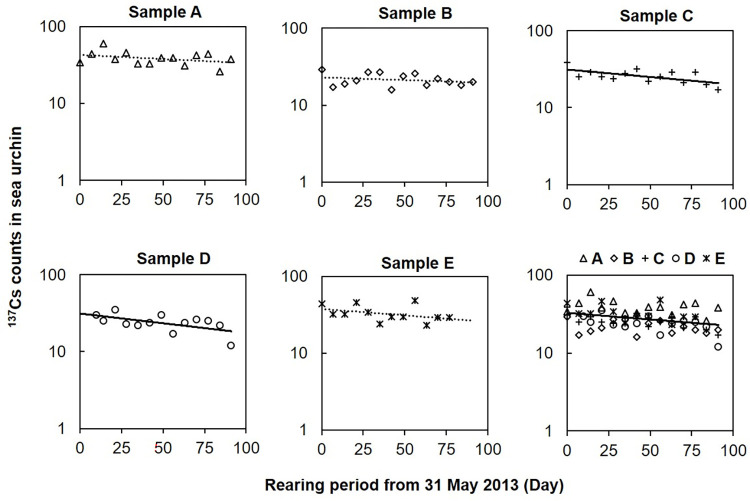
Temporal changes in the ^137^Cs counts in five sea urchins during the rearing period (31 May–30 August 2013) in laboratory conditions. The solid and dashed lines represent statistically significant and insignificant regression slopes, respectively.

The ^137^Cs counts were measured in five sea urchins during their rearing period (0–91 days) in the laboratory. The ^137^Cs counts in the sea urchins were between 29 and 44 at the start of the rearing period, and then decreased gradually until day 77–91 (Fig 2). We plotted the best fit exponential trend lines for the ^137^Cs counts in the five sea urchins (A–E) and listed the corresponding probabilities (*p*) in Table 1. The decreasing slopes of the ^137^Cs counts over time were significant in two of the five sea urchins (Pearson’s correlation coefficients of −0.601 for sample C (*p* < 0.05) and −0.581 for sample D (*p* < 0.05)). The decreases in the ^137^Cs counts for samples A, B, and E were not significant (*p* = 0.220, 0.324 and 0.218, respectively).

**Table 1:** Summary statistics for the regression slopes of the ^137^ Cs radioactivity (total number of ^137^ Cs counts), effective half-life (*T*_eff_, day), and biological half-life (*T*_b_, day) of all 5 individuals.

Sample ID	Coefficient of determination (*R*^*2*^)	*p*-value	Coefficient^a^		Effective half-life, *T*_ₑff_ (day)	Biological half-life, *T*_bio_ (day)
			*C*_0_ (Counts)	*λ*_bio_ (d^-1^)		
**A**	0.12	0.220	42.80	-0.00243	-	-
**B**	0.06	0.324	22.98	-0.01617	-	-
**C**	0.39	0.023*	31.30	-0.00447	155	157
**D**	0.34	0.038*	31.27	-0.00580	120	121
**E**	0.17	0.218	37.76	-0.00386	-	-
**All samples**	0.12	0.007*	32.78	-0.00389	178	181

*Statistical significance (*p* < 0.05) of the decreasing trend in the data for the reared sea urchin individuals.

^a^Coefficient for the following exponential equation for the measured data: *C*_t_ = *C*_0_e^(-*λ*^_bio_^t)^. *C*_t_ represents the ^137^Cs counts at elapsed day *t*. *C*_0_ represents the ^137^Cs counts at the time of the first measurement (31 May 2013) and *λ*_bio_ is the depuration rate constant.

The *T*_bio_ of ^137^Cs in sea urchin samples C and D that were reared for about 3 months in the laboratory were estimated as 157 and 121 days, respectively (Table 1). The ^137^Cs counts of samples A–E were significantly correlated with the elapsed days and plotted along a single trend line (*r* = −0.326 and *p* < 0.05). We plotted the 5 samples on a single plot and estimated the *T*_bio_ of ^137^Cs as 181 days (Fig 2).

The body weights (g) of the individual sea urchins were plotted against the rearing period (S2 Fig). There were significant correlations observed between the body weights of samples A, B, and D (*p* < 0.05) and the rearing period, but not between the body weights of samples C and E and the rearing period (*p* > 0.05). The specific growth rate (SGR) of the sea urchins was calculated as the percentage per day over a given time interval using the equation of Mahmud et al. (2016) [24], as follows:

$$Specific\;growth\;rate\;(\%,\;day^{-1})=(lnW_{2}–lnW_{1})/(d_{2}–d_{1})\times 100,$$ (5)

where W_2_ is the live body weight (g) at day d_2_, and W_1_ is the live body weight (g) at day d_1_.

The reduction rate in the ^137^Cs counts (*D*, day^−1^) was calculated with the formula used by Matsumoto et al. (2015) [15], as follows:

$$D=C_{n}-C_{n+1}/C_{n}(d_{n}-d_{n+1}),$$ (6)

where *C*_n_ and *C*_n+1_ were the ^137^Cs counts for the *n*th and *n*th + 1 measurements, respectively, and d_n_ and *d*_n+1_ were the number of days that elapsed between the *n*th and *n*th + 1 measurements when the initial time was set to 0 days.

The results from the generalized linear model (GLM) showed that the specific growth rate (SGR) of the sea urchins was significantly related to the rate of decrease in the ^137^Cs counts (*D*, day^−1^). The sample ID (A–E), elapsed days, and the SGR (%) per day were used as the explanatory variables in the GLM and the Akaike information criterion (AIC) was used to select the best model. The ^137^Cs counts reduction rate (*D*) per day in the sea urchins was the response variable. The result of a maximum likelihood test showed that the SGR of the sea urchins had a significant effect on *D* (ΔAIC = 1.85, χ^2^ = 8.72, *p* < 0.05) (S3 Table).

In S2 Fig, the relationships between the body weight and the rearing period of the individual sea urchin samples were depicted, and the growth tendency of only one sample (D) showed an opposite trend, and so had little effect on the GLM results. We used the data from all the samples in the GLM model to explore how the SGR affected the reduction rate (*D*) in the Cs counts during the rearing experiment. The GLM model showed that the ^137^Cs counts decreased significantly as the body growth of the sea urchin increased.

### Ecological half-life of radiocesium in the sea urchins

**Fig 3:**
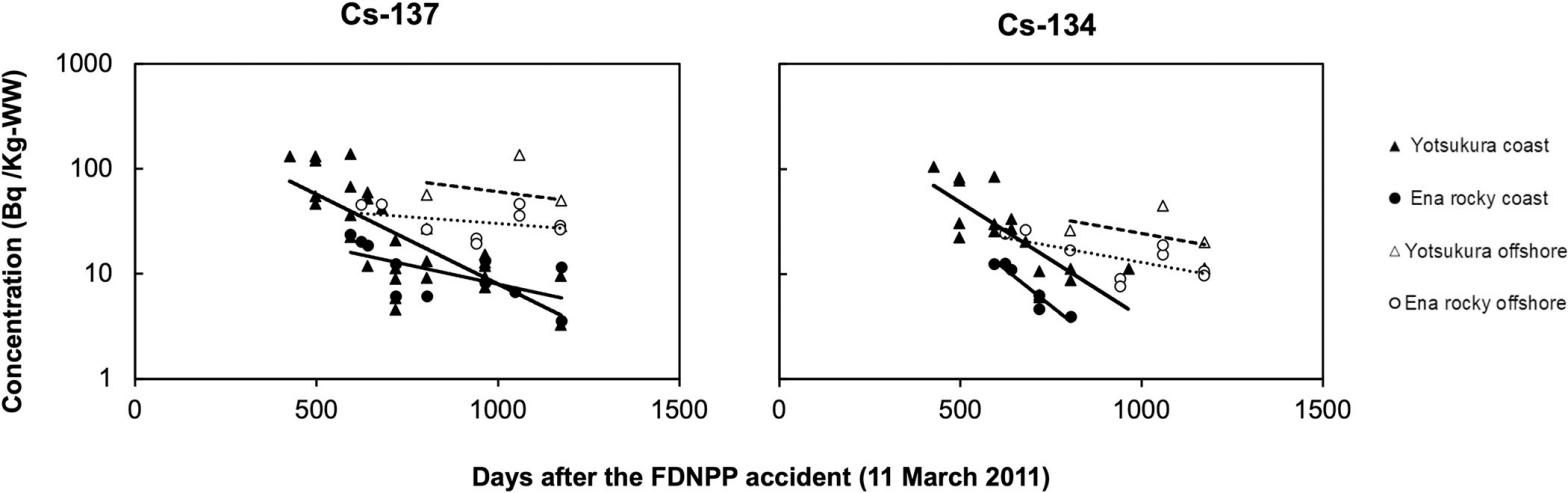
Spatial and temporal changes in the ^137^Cs and ^134^Cs activity concentrations in sea urchin gonads collected from four fishing grounds in Fukushima Prefecture after the FDNPP accident. Solid and dashed lines represent statistically significant and insignificant regression slopes, respectively. Different trend lines indicate different sampling stations for ^137^Cs (—Yotsukura coast,—Ena rocky coast,—Yotsukura offshore, and ⏤ · Ena rocky offshore) and ^134^Cs (—Yotsukura coast,—Ena rocky coast,—Yotsukura offshore, and ⏤ · Ena rocky offshore), respectively. Data below the detection limit and dry weight (DW) basis are excluded.

We determined the concentrations of radiocesium in sea urchins from four fishing areas (Yotsukura coast, Yotsukura offshore, Ena rocky coast, and Ena rocky offshore) near the FDNPP in Fukushima prefecture. As shown in Fig 3, the ^137^Cs and ^134^Cs activity concentrations (converted to log scale) in the sea urchin gonads tended to decrease over time (426–1175 days after the FDNPP accident). Between 500 and 600 days after the FDNPP accident in 2012, the radiocesium (^137^Cs and ^134^Cs) activity concentrations in the gonads of sea urchins collected from the Yotsukura coastal area peaked at 139 and 105 Bq/kg-WW, respectively. The average ^137^Cs activity concentrations in the gonads of sea urchins collected from the Yotsukura coast, Yotsukura offshore, Ena rocky coast, and Ena rocky offshore area in 2013 and 2014 were 14.3, 56.5, 9.19, and 28.4 Bq/kg-WW, and 6.41, 71.1, 7.24, and 34.3 Bq/kg-WW, respectively. Between 2012 and 2014, the decrease in the radiocesium (^137^Cs and ^134^Cs) activity concentrations in the gonads of the sea urchins was greater in those from the coast areas (e.g. solid lines) than in those from the offshore areas (e.g. dashed lines in Fig 3). Both radiocesium (^137^Cs and ^134^Cs) activity concentrations in the gonads of sea urchins collected from the Yotsukura coast and Ena rocky coast decreased significantly (*p* < 0.05) (Table 2). By 2726 days after the FDNPP accident, the ^137^Cs activity concentrations were 0.13–0.17 Bq/kg-DW and ^134^Cs was not detected in the gonads of sea urchins collected from the Yotsukura coast site (S2 Table).

**Table 2:** Summary statistics of the ^137^Cs and ^134^Cs radioactivity, effective half-life (*T*_eff_, day), and ecological half-life (*T*_eco_, day) in sea urchin gonads collected from the four sampling points (Yotsukura coast and offshore areas, and Ena rocky coast and offshore areas).

Study area	Radiocesium	Coefficient of determination (*R*^*2*^)	*p*-value	Coefficient^a^		Effective half-life (*T*_ₑff_) days	Ecological half-life (*T*_eco_) days
				*C*_0_ (Bq/kg)	*λ*_eco_ (d^-1^)		
**Yotsukura coast**	^137^Cs	0.62	<0.01^*^	407.3	-0.0039	178	181
	^134^Cs	0.61	<0.01^*^	595.9	-0.0050	139	170
**Yotsukura offshore**	^137^Cs	0.01	0.26	166.1	- 0.0010	-	-
	^134^Cs	0.07	0.26	99.36	-0.0014	-	-
**Ena rocky coast**	^137^Cs	0.51	0.03^*^	44.19	- 0.0017	408	423
	^134^Cs	0.92	<0.01^*^	584.46	-0.0064	108	126
**Ena rocky offshore**	^137^Cs	0.18	0.72	54.72	-0.0006	-	-
	^134^Cs	0.56	0.16	54.15	-0.0014	-	-

*The decreasing trend was statistically significant (*p* < 0.05).

^a^ Coefficient for the following exponential equation for the measured data: *C*_t_ = *C*_0_e^(-*λ*^_eco_^t)^. *C*_t_ represents the ^137^Cs and ^134^Cs concentrations at elapsed day *t*, *C*_0_ represents the ^137^Cs and ^134^Cs concentrations at the time of first measurement, and *λ*_eco_ represents the decay rate constant.

The decreases in the slopes of the activity concentrations of both radionuclides in the sea urchins collected from the Yotsukura coast area were significant (*p* < 0.05) (Table 2). The *λ*_eco_ of ^137^Cs in the gonads of sea urchins was higher in the Yotsukura coast (0.0039 d^−1^) than in the samples from the other stations. Highest decay rate constant of ^134^Cs in sea urchin gonad was obtained at Ena rocky coast (*λ*_eco_ = 0.0064 d^−1^). The *T*_eco_ and *T*_eff_ of ^137^Cs and ^134^Cs in the gonads of sea urchins were estimated from the samples collected from the Fukushima coast and offshore areas (Table 2). The *T*_eff_ and *T*_eco_ of ^137^Cs in the sea urchins from the Yotsukura coastal area were calculated as 178 days and 181 days, respectively. Longer *T*_eff_ and *T*_eco_ were observed at Ena rocky coast (408 and 423 days, respectively). The *T*_eff_ and *T*_eco_ were not determined for the other stations as the ^137^Cs activity concentrations did not decrease significantly with time.

The *T*_eff_ and *T*_eco_ for ^134^Cs in the gonads of sea urchins from the Yotsukura coast and Ena rocky coast were 139 and 170 days, and 108 and 126 days, respectively, and the activity concentrations decreased significantly (*p* < 0.05) in the period since the FDNPP accident. We did not determine the *T*_eff_ and *T*_eco_ in the sea urchins from the Yotsukura offshore and Ena rocky offshore stations, as the decreases in the ^134^Cs activity concentrations in the sea urchin gonads from the Yotsukura offshore and Ena rocky offshore stations (*p* = 0.26 and 0.21, respectively) were not significant during the survey period.

The half-life of radioactive Cs was determined from the whole body for *T*_bio_ and from the gonad for *T*_eco_. For this comparison, the activity concentrations of both were measured at the same time. The ^137^Cs activity concentrations in the whole bodies and gonads at the Yotsukura coast were ND–5.9 and 11.9–59.4 Bq/kg-WW in December 2012, ND–4.1 and 4.56–20.8 Bq/kg-WW in February 2013, and 5.06–7.94 and 3.26–9.56 Bq/kg-WW in May 2014, respectively (S2 Table).

### ACF in sea urchin gonads

The ^137^Cs activity concentrations in seawater at the Yotsukura coast and Ena rocky coast stations were 0.01–0.16 Bq/L and 0.01–0.10 Bq/L, respectively (S3 Fig). The regression slopes for ^137^Cs activity in seawater from the Yotsukura and Ena stations were significant (*p* < 0.05) (S4 Table). The depuration rate constants (*λ*) of ^137^Cs in seawater and in the gonads of sea urchins from the Yotsukura coast and Ena rocky coast stations were 0.0011 and 0.0015 d^−1^ and 0.0039 and 0.0022 d^−1^, respectively, so the values in the seawater were lower than those in the gonads. Moreover, the *T*_eco_ values of ^137^Cs in seawater from the Yotsukura and Ena stations were 681 and 487 days, respectively, and were greater than the value for the gonads of sea urchins (181 days) at the Yotsukura coast.

**Fig 4:**
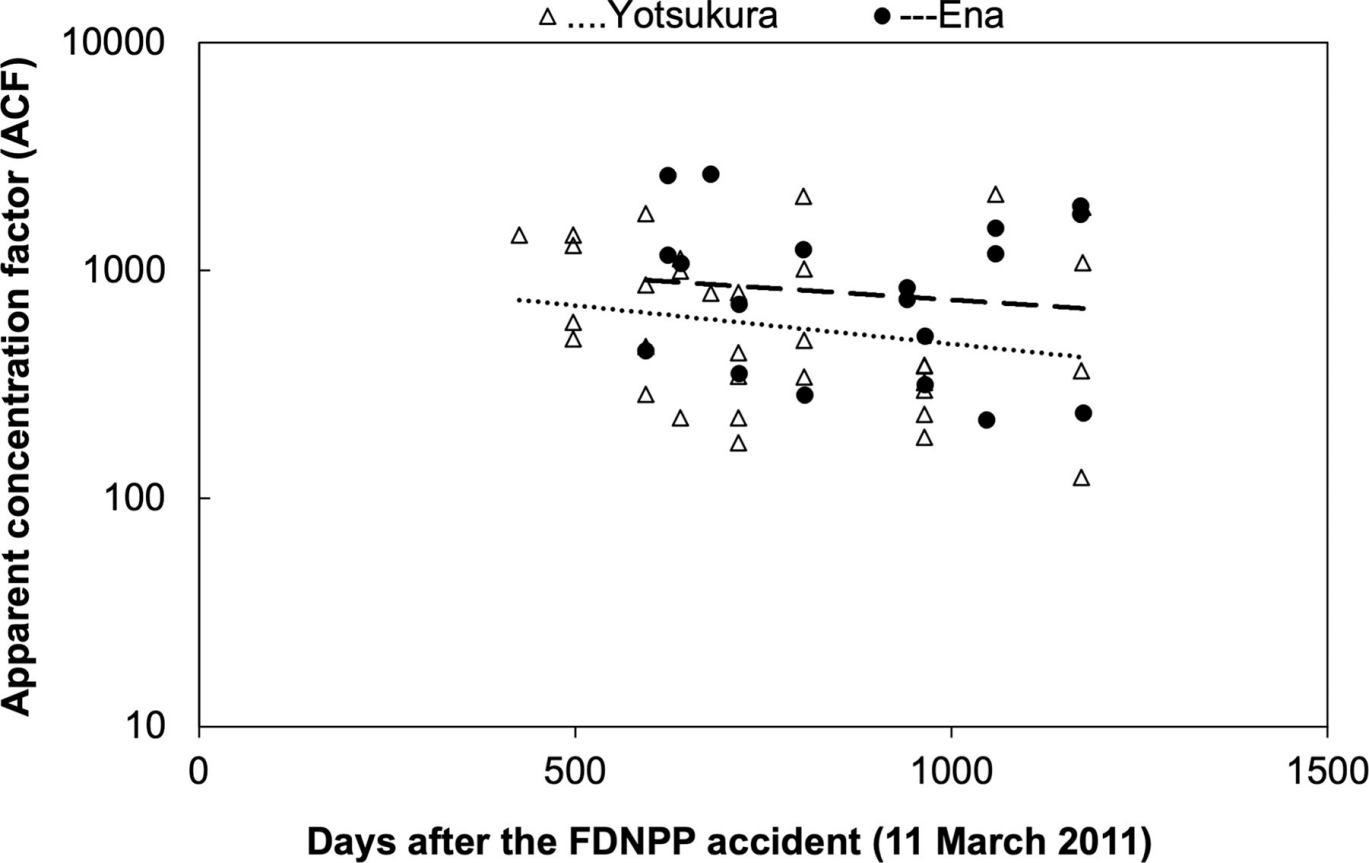
Spatial and temporal variations in the apparent concentration factor (ACF) of ^137^Cs in sea urchins. Dashed lines represent statistically insignificant regression slopes.

The ACF in the gonads of sea urchins from the Fukushima coastal area varied widely (123.8–2648) between 426 and 1175 days after the FDNPP accident (Fig 4). The ACF of ^137^Cs in the gonads of sea urchins from the Yotsukura and Ena stations showed a decreasing trend, but the ACF was not significantly correlated with the number of days since the accident at either the Yotsukura stations (*r*_*s*_ = −0.24, *p* = 0.18) or the Ena stations (*r*_*s*_ = −0.10, *p* = 0.69). The ACF values at the Yotsukura and Ena stations had ranges of 123.8–2163 and 220.9–2648, and median values of 493.5 and 840.3, respectively. At the Yotsukura coast site, 2726 days after the FDNPP accident, the ACF was 118.0±15.99.

## Discussion

### Radiocesium contamination after the FDNPP accident

Individuals of *M*. *nudus* were collected from the Ena coastal area on May 2013 after the FDNPP accident and were cultured in a water tank for up to 91 days. Our results showed that the ^137^Cs counts decreased significantly in two of the five individuals. As shown in S2 Fig, the body weights of samples A and B increased, but the body weight of sample D decreased (*p* < 0.05). We are not sure what caused the weight loss in sample D. The spawning season for sea urchins in the target sea area (off the coast of Fukushima Prefecture) is from July to August [25] and our rearing experiment was carried out between May and August, thus the sea urchin individuals may have spawned during the experimental period. Consequently, reared sea urchins may gain weight in the first half of summer and then lose weight due to spawning. Sample D, therefore, was in the weight loss phase in the latter half of summer. We did not confirm whether the samples were males or females, so we cannot say if there was a gender effect. The weight loss may have been related to the release of germ cells, a decrease in food intake because of an increase in the water temperature during summer, or stress in the breeding environment.

The decreases in the ^137^Cs counts were significant in some sea urchin samples (C and D) but were insignificant in other samples (Fig 2). The trends in the body weights of the individual sea urchins and the ^137^Cs counts were not uniform. To explore the relationship between the growth and the ^137^Cs counts in the sea urchins, the specific growth rates (SGR, % day^−1^) of sea urchins and reduction rates (*D*, day^−1^) in the ^137^Cs counts were calculated, and then a multivariate statistical model (GLM) was built using the data of all (five) sea urchin samples. The results of the GLM model showed that the SGR of the sea urchins was significantly related to the reduction rate in the ^137^Cs counts, i.e., when the growth rate of a sea urchin increased, then the reduction rate in the ^137^Cs counts also increased in our rearing environment. Other studies of different marine organisms showed that the body size had a significant effect on the depuration rate of ^137^Cs [15, 26, 27].

It is thought that any decreases in the variables measured in the sea urchins from the sampled sea areas reflect the habitats. In this study, the *T*_bio_ was determined from the decreasing tendency in the overall ^137^Cs counts. When the data for all five individuals are included, the excretion rate coefficient *λ*_bio_ was 0.00389 d^−1^ and the *T*_bio_ was 181 days (Table 1). This result, calculated from the data of all the individuals, was used as the excretion rate coefficient of ^137^Cs of *M*. *nudus* and agrees well with the results from Aomori Prefecture (1988) [28], who reported an excretion rate coefficient of 0.0042 d^−1^ for ^137^Cs of *M*. *nudus* taken from the food.

The radiocesium (^137^Cs and ^134^Cs) activity concentrations in sea urchins *M*. *nudus* were monitored for the period between 426 and 2726 days after the FDNPP accident (11 March 2011). The maximum ^137^Cs activity concentration in the gonads of sea urchins on 25 October 2012, at 139 Bq/kg-WW, was greater than the Japanese regulatory limit of radiocesium for foodstuffs (100 Bq/kg-WW), but was lower than the maximum value (290 Bq/kg-WW) reported in sea urchins by Sohtome et al. (2014) [17]. The activity concentrations decreased gradually over time in the area close to the FDNPP, such that, in 2018, the ^137^Cs activity concentrations in the gonads of sea urchins were 0.13–0.17 Bq/kg-DW), and ^134^Cs was not detected at the Yotsukura station (S2 Table).

The ^137^Cs activity concentrations in *M*. *nudus* in the Fukushima coastal area were examined, and the *T*_eco_ was calculated from the *λ*_eco_. The highest activity concentration of radiocesium was observed at Yotsukura coast. The trendline of the temporal changes in the ^137^Cs activity concentrations at the coast areas (Yotsukura and Ena rocky) showed a significant exponential correlation (*p* < 0.05) (Table 2). The *λ*_eco_ and *T*_eco_ of ^137^Cs at the Yotsukura coast were 0.0039 d^−1^ and 181 days, respectively, and were 0.0017 d^−1^ and 423 days at the Ena rocky coast, respectively. Iwata et al. (2013) reported a value of 126±12 days for the *T*_eco_ of ^137^Cs in *M*. *nudus* [16]. Their results were derived from over 1 to 2 years, and so are useful for understanding how the ^137^Cs activity concentrations decreased in the period immediately after the accident. Our results present the changes in the ^137^Cs activity concentrations between 2 and 4 years after the accident. The difference in the study period affects the *T*_eco_. The *T*_eco_ of ^137^Cs was 181 days at the Yotsukura coast sampling point, which was close to the FDNPP. It was reported that, during the FDNPP accident, highly contaminated water flowed directly out of the plant, diffused, and flowed to the south [3]. After the highly contaminated water had passed, the ^137^Cs activity concentrations in seawater immediately decreased (< 0.10 Bq/L) [29, 30]. Therefore, we infer that the organisms near the FDNPP were in contact with highly contaminated water. Thus, the spatial differences in the *T*_eco_ may be a legacy of the initial contamination.

The *T*_eco_ of ^137^Cs of the sea urchin from the Ena rocky coast (423 days) was much longer than that of the sea urchin from the Yotsukura coast (181 days) (Table 2). The faster depuration rate of ^137^Cs in the sea urchin from Yotsukura may reflect the ongoing gradual decreases in the ^137^Cs activity concentrations in seawater and sediment at the Yotsukura station (S3 Fig). Also, the difference between the *T*_eco_ values may be attributable to the difference in the distance of the sampling stations from the FDNPP (Fig 1), number of samples analyzed, amount of ^137^Cs deposited on the sediments, and the uptake of ^137^Cs contaminated food. Matsumoto et al. (2018) reported that the residual half-life (*T*_re_) of ^137^Cs was much longer in sediments collected from the Ena rocky station (736 days) than in those from Yotsukura (482 days) [12]. This may help explain the longer *T*_eco_ of ^137^Cs in the sea urchin caught from the Ena rocky coast of Fukushima. Because the organic matter in sea sediment contains high ^137^Cs that would gradually translocate to the lithogenic fraction [14] which might be ingested by sea urchin. In another study, Shigeoka et al. (2019) reported that the ^137^Cs activity concentrations in some seagrasses, which are important food source for sea urchins, were higher at Ena station than at the Yotsukura station [9]. Thus, the spatial difference in the *T*_eco_ might be attributed by deposited ^137^Cs in sediment which was transferred through food uptake (e.g., organic matter and marine plants) by the sea urchins.

From their sea urchin rearing experiment, Nakamura et al. (1986) reported that each sea urchin had a unique distribution of radionuclides in the shell, spine, digestive tract, and gonad [31]. They also clarified that the whole-body (spine and shell) contamination of a sea urchin was mainly from radionuclides in seawater, and that the contamination of the digestive tract and gonad was from both seawater and food uptake. The ^137^Cs activity concentrations in seawater decreased very quickly after the FDNPP accident, so the concentrations were low during the survey period (< 1 Bq/L) (S3 Fig) and the ^137^Cs activity concentrations were low in the whole body of the sea urchins. However, the ^137^Cs activity concentrations in the food of sea urchins contributed significantly to the ^137^Cs accumulation in the gonad [31], which may explain why the ^137^Cs activity concentrations were higher in the gonad samples than in whole body samples.

At the beginning of this survey, the ^137^Cs activity concentrations in the sea urchins were much higher in the coastal samples than in the offshore samples (Fig 3). The ^137^Cs activity concentrations decreased significantly in the coastal samples over the period, but the ^137^Cs activities did not change significantly in the offshore samples (Table 2). In contrary, the average ^137^Cs activity concentrations in the sea urchins collected from the Yotsukura offshore (67.5±46.6 Bq/kg-WW) and Ena rocky offshore areas (31.6±10.9 Bq/kg-WW) were higher than those of coastal areas (Yotsukura = 37.9±41.8 Bq/kg-WW, Ena = 10.9±6.65 Bq/kg-WW) but the spatial differences were insignificant (ANOVA; *p* = 0.053). These results indicate that the ^137^Cs activity concentrations in sea urchins might be influenced by the consumption of ^137^Cs contaminated food by sea urchins, and may have been related to the ingestion of organic matter in seabed sediment with high ^137^Cs activity concentrations in the offshore area to the south of the FDNPP [10, 11]. Additionally, the high ^137^Cs in sea urchins may be related to the high ^137^Cs activity concentrations in a rocky reef in the offshore area, as reported by Suzuki et al. (2019) [32].

The *T*_bio_ was determined from the cesium counts in the whole body, and the *T*_eco_ was determined from the cesium activity concentrations in the gonad. As mentioned in the results section (line 336–339), the ^137^Cs activity concentrations in the gonads tended to be high relative to those in the whole body until 2013, but were similar in 2014. Very little cesium had accumulated in the whole body until 2013. This suggests that the change in the concentrations of radioactive substances in the whole body was due to the change in the radioactivity in the gonads; that the samples were from different parts of the sea urchins had little effect on the half-life.

The *T*_bio_ values of ^137^Cs of the individual sea urchin samples were between 121 and 157 days, while the value for all the reared samples was 181 days (Table 1). The *T*_bio_ values were similar or close to the *T*_eco_ value (181 days) of ^137^Cs determined from the gonads of the sea urchins at the Yotsukura coast but shorter than the value for the Ena coast (423 days). It was also revealed that there was a significant difference (ANCOVA, *p* < 0.0001) between the lab experiment and the field sample (Ena and Yotsukura coasts) (S5 Table).

We compiled information about the ^137^Cs activity concentrations in seawater (Bq/L), marine plants (Bq/kg), and sediments (Bq/kg) reported for the period from April 2012 to May 2014 by Shigeoka et al. (2019) and Matsumoto et al. (2018) [9, 12]. We compared their values with those from this study and plotted exponential trend lines over time in a depuration graph of the ^137^Cs activity concentrations (natural log scale) in the gonads of sea urchins, seawater, marine plants (brown algae, red algae, and seagrass), and sediment at the Yotsukura and Ena stations (S3 Fig). The ^137^Cs activity concentrations at both sampling stations were ranked as follows: sediment > sea urchin > marine plants > seawater.

The apparent concentration factor (ACF) measured in our study was much higher (123.1–2648) than the values reported in previous studies (ACF = 10–20) [28, 31]. The ACF was high after the accident, and then decreased gradually, but the decrease was not significant (Fig 4). The ACF value was still high (118.0±15.99) compared with the previously reported values even 2726 days after the accident, when the ^137^Cs activity concentrations in seawater were low [31]. Thus, in 2018, the sea urchin ^137^Cs activity concentrations were probably related to uptake from seawater and food.

Cesium is mainly ingested into sea urchins through food and water, and the food can have more effect on the accumulation of ^137^Cs than the seawater [31]. Types of kelp or seaweed, the main foods of sea urchins, contribute to the contamination in the edible part of sea urchins. Of 16 species of marine plants contaminated by radiocesium [9], 2 species (*Eclonia bicyclis* and *Phyllospadix iwatensis*) may have contributed to the contamination of sea urchins when ingested [21, 31]. The uptake of ^137^Cs will differ depending on the species of seaweed consumed as food. Tateda et al. (2013) reported that the ^137^Cs depuration rate was slower for sea urchins (by 2.1 times) than for other algae feeding invertebrates, because of the contribution of contaminated food (suspended particles attached to the algae and algal fragments) in their digestive tract [33].

### Causes of long term ^137^Cs contamination in sea urchins

In this study, the biological half-life and the ecological half-life were determined simultaneously, so we were able to calculate the ^137^Cs activity concentration in the food of *M*. *nudus* collected from the Fukushima coast over the study period. Here, we adopted a compartment model for primary consumer herbivorous invertebrates [34], shown in Eq (7), and used the *λ*_bio_ and *λ*_eco_ values from this study (Tables 1 and 2) to calculate the ^137^Cs concentration in the food of *M*. *nudus* in 1 day.

$$dB_{2(t)}/dt=k_{02}S_{\left(t\right)}+k_{12}B_{1(t)}‐k_{20}B_{2(t)},$$ (7)

where *S*_(t)_ is the ^137^Cs concentration in seawater (Bq/L), and *B*_1(t)_ and *B*_2(t)_ indicate the ^137^Cs concentrations of a producer and a primary consumer (Bq/kg-WW), respectively. *t* is the time (days). *k*_02_, *k*_20_, and *k*_12_ denote the uptake rate coefficient of the primary consumer from seawater (d^−1^), excretion rate coefficient (d^−1^), and the uptake rate coefficient from food (d^−1^), respectively.

Eq (7) was therefore transformed into a formula for the ^137^Cs concentration of food, (Eq (7)′)

$$B_{1(t)}=(dB_{2(t)}/dt-k_{02}S_{\left(t\right)}+k_{20}B_{2(t)})/k_{12},$$ (7)′

d*B*_2(t)_/d*t* was calculated from B_2_ (t + 1) − B_2_ (t) * exp (−*λ*_eco_). The values of *k*_02_ and *k*_12_ were 0.1568 and 0.0042, respectively [28]. *k*_20_ was set to *λ*_bio_; 0.003889, i.e., the result from this study. The ^137^Cs activity concentrations in the food of sea urchins at the Yotsukura station up to 700 days after the accident and at about 1000 days after the accident were estimated as 116–19037 Bq/kg and 4483–31417 Bq/kg, respectively (S4 Fig).

Various researchers have reported the concentrations of ^137^Cs in seaweed along the Fukushima coast after the accident [9, 14, 16]. Shigeoka et al. (2019) investigated the ^137^Cs activity concentrations in 15 species of algae and 1 seagrass species and found that the ^137^Cs activity concentrations in algae at 500 and 1000 days after the accident were 2.86–12.3 Bq/kg-WW and 0.22–0.58 Bq/kg-WW, respectively (S3 Fig) [9]. The ^137^Cs activity concentration in food calculated in this study (S4 Fig) was 20–2554 times higher than the concentration in algae. These results therefore suggest that the ^137^Cs contamination in the sea urchins was not exclusively from seaweed but was also from other food consumed.

As sea urchins are bottom dwelling invertebrates, higher ^137^Cs activity concentrations in sea sediment (S3 Fig) may transfer to them through benthic food web. Otosaka and Kobayashi (2013) demonstrated that organically bound ^137^Cs in sediment collected from the coastal area 70 km south of the FDNPP contributed about 20% of sedimentary ^137^Cs, even though the sediment had a relatively low proportion of organic matter (4–6%) [35]. These results suggest that the bioavailable fraction (e.g., detritus) of ^137^Cs in sediment is an ongoing source of radioactive cesium to the sea urchin.

Suzuki et al. (2019) showed that the ^137^Cs activity concentrations on reefs were still high after the accident, which suggests that the high levels of ^137^Cs are sustained by ongoing contamination [32]. Sea urchins also eat coralline red algae that adhere to the rocky reef. There are no published investigations of the concentrations of radioactive substances in coralline, so we can reasonably suspect that some foods of sea urchin are still severely contaminated. It has been reported that about 3–12% of the seabed sediments is organic matter [12]. Sea urchins, therefore, eat organic matter in the sediments with unknown radiocesium concentrations and seaweed, resulting in a long ecological half-life.

In this study, we investigated the temporal changes in the ^137^Cs activity concentrations in the sea urchin *M*. *nudus* after the FDNPP accident and determined the biological and ecological half-lives. From these results, we examined the relationship between the changes in the ^137^Cs activity concentrations in the marine environment after the accident and the sea urchin contamination. We found that the changes in the ^137^Cs activity concentrations in the sea urchins reflected the decrease in the ^137^Cs activity concentrations in the food rather than the decrease in the seawater concentrations. These results reinforce that, to reduce the concentrations of radioactive materials in organisms in the Fukushima area, the concentrations of radioactive materials in ecosystems, including those in prey organisms, must first decrease.

## Conclusions

After the Great East Japan Earthquake in 2011, radiocesium was dispersed into the ocean environment near the FDNPP. In this study, radiocesium contamination of the sea urchin *M*. *nudus* reared in laboratory and field conditions was monitored. The *T*_bio_ and *T*_eco_ of radiocesium were also ascertained. The *T*_bio_ values of ^137^Cs in individual sea urchins reared in the laboratory were between 121–157 days and were shorter than the *T*_eco_ of ^137^Cs in gonads from sea urchins collected from the marine environment (181–423 days). The *T*_eco_ measurements reflect direct contamination by radiocesium from the surroundings (e.g., water, sediment) and food habits (e.g., kelp, seaweed). The ^137^Cs activity concentrations in the sea urchins reflect the ^137^Cs activity concentrations in the food rather than the seawater radioactivity. However, we did not identify the type of food. It would be useful to carry out further studies of the *T*_eco_ and *T*_bio_ of various species to improve our understanding of how seafood ingest and eliminate ^137^Cs.

## Supporting information

S1 Table Shell length of sea urchin samples at the beginning of the rearing experiment. (DOCX)

S2 Table ^137^Cs and ^134^Cs activity concentrations measured in sea urchins from the Fukushima area. (DOCX)

S3 Table Results from GLM analysis of the ^137^Cs counts reduction rate (*D*) per day in sea urchins, and a maximum likelihood test against the null model for the specific growth rate of the sea urchins. AIC = −220.05.
(DOCX)

S4 Table Summary statistics for the regression slopes of the ^137^Cs radioactivity (total number of ^137^Cs concentration), effective half-life (*T*_eff_, day), and ecological half-life (*T*_eco_, day) in seawater from the Yotsukura and Ena stations. (DOCX)

S5 Table Analysis of covariance (ANCOVA) for the relative value of the ^137^Cs activity (natural log scale) changes that were used to calculate the half-lives (*T*_bio_ and *T*_eco_) in sea urchins. (DOCX)

S1 Fig Trend of ^137^Cs activity concentrations (Bq/L; Mean±SD) in seawater during the rearing experiment of sea urchins (Kaeriyama, 2017). (DOCX)

S2 Fig Temporal changes in the body weights of sea urchins during the rearing period (31 May–30 August 2013) in the laboratory. Solid and dashed lines represent statistically significant and insignificant regression slopes, respectively.
(DOCX)

S3 Fig Spatial and temporal changes in the ^137^Cs activity concentrations in sea urchin gonads (Bq/kg-WW), seawater (Bq/L), marine plants (Bq/kg-WW), and sediment (Bq/kg-DW) collected from two fishing areas in the Fukushima Prefecture after the FDNPP accident. Filled and empty squares in the water data show our survey values and the NRA data, respectively. Solid lines indicate fitted exponential functions for ^137^Cs concentrations in sea urchins, seawater, marine plants, and sediment. Data below the detection limit were excluded.
(DOCX)

S4 Fig Uptake of ^137^Cs through food in sea urchins (Bq/Kg-WW) collected from the Yotsukura fishing ground in the Fukushima Prefecture after the FDNPP accident. (DOCX)
