# Supplementary material for: Contamination of sea urchin Mesocentrotus nudus by radiocesium released during the Fukushima Daiichi Nuclear Power Plant accident
Source: PLoS One. 2022 Aug 15;17(8):e0269947. doi: 10.1371/journal.pone.0269947 (PMC9377606; doi:10.1371/journal.pone.0269947)
Supplement: S1 Table — (DOCX) [file pone.0269947.s001.docx]

**S1 Table. Shell length of sea urchin samples at the beginning of rearing experiment.**

| **Sample name** | **Shell length (mm)** |
| --- | --- |
| A | 54.1 |
| B | 54.8 |
| C | 57.2 |
| D | 59.7 |
| E | 53.7 |
| Average (±SD) | 55.4 ± 2.5 |
