# Supplementary material for: Contamination of sea urchin Mesocentrotus nudus by radiocesium released during the Fukushima Daiichi Nuclear Power Plant accident
Source: PLoS One. 2022 Aug 15;17(8):e0269947. doi: 10.1371/journal.pone.0269947 (PMC9377606; doi:10.1371/journal.pone.0269947)
Supplement: S2 Table — (DOCX) [file pone.0269947.s002.docx]

**S2 Table. ^137^Cs and ^134^Cs activity concentrations measured in sea urchins from the Fukushima area.**

| **Sample No.** | **Sampling Date (Day / Month / Year)** | **Sampling site** | **Passing days after the accident** | **Sample weight (Kg)** | **Cs-134 (Bq/Kg-WW)** | **Detection limit of Cs-134** | **Cs-137 (Bq/Kg-WW)** | **Detection limit of Cs-137** | **Category of sample** |
| --- | --- | --- | --- | --- | --- | --- | --- | --- | --- |
| 1 | 10/5/2012 | Yotsukura Coast | 426 | 0.09 | 105 | 13.1 | 132 | 10.8 | Gonad |
| 2 | 20/7/2012 | Yotsukura Coast | 497 | 0.1 | 77.6 | 10.7 | 132 | 9.72 | Gonad |
| 3 | 20/7/2012 | Yotsukura Coast | 497 | 0.098 | 82.6 | 10.2 | 119 | 9.1 | Gonad |
| 4 | 20/7/2012 | Yotsukura Coast | 497 | 0.095 | 22.3 | 11.1 | 46.3 | 7.68 | Gonad |
| 5 | 20/7/2012 | Yotsukura Coast | 497 | 0.102 | 30.6 | 10.2 | 54.9 | 11.3 | Gonad |
| 6 | 25/10/2012 | Yotsukura Coast | 594 | 0.048 | 29.9 | 12.1 | 67.6 | 11 | Gonad |
| 7 | 25/10/2012 | Yotsukura Coast | 594 | 0.053 | 25.5 | 13 | 36.3 | 10.6 | Gonad |
| 8 | 25/10/2012 | Yotsukura Coast | 594 | 0.027 | 84.3 | 20.8 | 139 | 16.1 | Gonad |
| 9 | 25/10/2012 | Yotsukura Coast | 594 | 0.037 | ND | 15.9 | 22.3 | 12.1 | Gonad |
| 10 | 25/10/2012 | Ena rocky Coast | 594 | 0.1 | 12.4 | 9.03 | 23.6 | 8.19 | Gonad |
| 11 | 23/11/2012 | Ena rocky Offshore | 623 | 0.1 | 24.1 | 3.96 | 45.4 | 4.4 | Gonad |
| 12 | 23/11/2012 | Ena rocky Coast | 623 | 0.101 | 12.6 | 3.45 | 20.2 | 3.72 | Gonad |
| 13 | 10/12/2012 | Yotsukura Coast | 640 | 0.052 | 33.3 | 10.1 | 59.4 | 13.1 | Gonad |
| 14 | 10/12/2012 | Yotsukura Coast | 640 | 0.094 | 27 | 9.29 | 52.2 | 7.59 | Gonad |
| 15 | 10/12/2012 | Yotsukura Coast | 640 | 0.083 | ND | 9.17 | 11.9 | 8.72 | Gonad |
| 16 | 10/12/2012 | Yotsukura Coast | 640 | 0.126 | ND | 2.86 | 5.94 | 2.93 | Whole Body |
| 17 | 10/12/2012 | Yotsukura Coast | 640 | 0.123 | ND | 3.45 | ND | 3.32 | Whole Body |
| 18 | 10/12/2012 | Yotsukura Coast | 640 | 0.123 | 4.17 | 2.44 | 4.6 | 2.53 | Whole Body |
| 19 | 10/12/2012 | Yotsukura Coast | 640 | 0.09 | ND | 7.99 | ND | 9.58 | Whole Body |
| 20 | 11/12/2012 | Ena rocky Coast | 641 | 0.095 | 11 | 7.93 | 18.6 | 7.37 | Gonad |
| 21 | 19/1/2013 | Yotsukura Coast | 680 | 0.08 | 20.2 | 4.4 | 41.8 | 4.88 | Gonad |
| 22 | 19/1/2013 | Ena rocky Offshore | 680 | 0.114 | 26.2 | 3.61 | 45.9 | 3.84 | Gonad |
| 23 | 25/02/2013 | Yotsukura Coast | 717 | 0.112 | ND | 2.95 | ND | 3.41 | Whole Body |
| 24 | 25/02/2013 | Yotsukura Coast | 717 | 0.113 | ND | 2.66 | ND | 3.49 | Whole Body |
| 25 | 25/02/2013 | Yotsukura Coast | 717 | 0.103 | ND | 4.11 | ND | 3.84 | Whole Body |
| 26 | 25/02/2013 | Yotsukura Coast | 717 | 0.089 | ND | 3.6 | 4.07 | 3.25 | Whole Body |
| 27 | 25/02/2013 | Yotsukura Coast | 717 | 0.094 | ND | 3.26 | ND | 3.08 | Whole Body |
| 28 | 25/02/2013 | Yotsukura Coast | 717 | 0.064 | 5.99 | 3.82 | 8.95 | 4.01 | Gonad |
| 29 | 25/02/2013 | Yotsukura Coast | 717 | 0.089 | ND | 3.14 | 5.85 | 3.8 | Gonad |
| 30 | 25/02/2013 | Yotsukura Coast | 717 | 0.089 | 10.7 | 3.73 | 20.8 | 3.57 | Gonad |
| 31 | 25/02/2013 | Yotsukura Coast | 717 | 0.091 | ND | 4.07 | 4.56 | 4.36 | Gonad |
| 32 | 25/02/2013 | Yotsukura Coast | 717 | 0.091 | 6.33 | 3.73 | 11.3 | 3.89 | Gonad |
| 33 | 26/2/2013 | Ena rocky Coast | 718 | 0.093 | 4.64 | 2.95 | 6.1 | 3.12 | Gonad |
| 34 | 26/2/2013 | Ena rocky Coast | 718 | 0.098 | 6.27 | 3.23 | 12.3 | 3.55 | Gonad |
| 35 | 26/2/2013 | Ena rocky Coast | 718 | 0.107 | ND | 3.97 | ND | 3.68 | Whole Body |
| 36 | 26/2/2013 | Ena rocky Coast | 718 | 0.108 | ND | 3.09 | ND | 3.45 | Whole Body |
| 37 | 22/5/2013 | Yotsukura offshore | 803 | 0.07 | 26 | 5.43 | 56.5 | 5.97 | Gonad |
| 38 | 22/5/2013 | Ena rocky Offshore | 803 | 0.062 | 16.9 | 5.57 | 26.5 | 6.52 | Gonad |
| 39 | 23/5/2013 | Yotsukura Coast | 804 | 0.093 | 8.7 | 3.81 | 13.2 | 4 | Gonad |
| 40* | 23/5/2013 | Yotsukura Coast | 804 | 0.038 | ND | 0.136 | 2.60 | 0.004 | Gonad |
| 41 | 23/5/2013 | Yotsukura Coast | 804 | 0.106 | ND | 3.46 | 9.11 | 3.94 | Gonad |
| 42 | 23/5/2013 | Yotsukura Coast | 804 | 0.061 | 11.2 | 4.38 | 27.1 | 4.36 | Gonad |
| 43 | 24/5/2013 | Ena rocky Coast | 805 | 0.022 | 3.91 | 2.08 | 6.11 | 1.91 | Gonad |
| 44 | 7/10/2013 | Ena rocky offshore | 941 | 0.082 | 9.05 | 3.64 | 21.7 | 3.88 | Gonad |
| 45 | 7/10/2013 | Ena rocky offshore | 941 | 0.081 | 7.63 | 2.86 | 19.3 | 3.38 | Gonad |
| 46 | 30/10/2013 | Yotsukura Coast | 964 | 0.024 | ND | 7.88 | 9.32 | 6.74 | Gonad |
| 47 | 30/10/2013 | Yotsukura Coast | 964 | 0.034 | 11.2 | 6.45 | 15.2 | 6.49 | Gonad |
| 48 | 30/10/2013 | Yotsukura Coast | 964 | 0.032 | ND | 7.28 | 15.3 | 7.36 | Gonad |
| 49 | 30/10/2013 | Yotsukura Coast | 964 | 0.06 | ND | 3.94 | 12.8 | 3.5 | Gonad |
| 50 | 30/10/2013 | Yotsukura Coast | 964 | 0.021 | ND | 7.96 | ND | 8.41 | Gonad |
| 51 | 30/10/2013 | Yotsukura Coast | 964 | 0.103 | ND | 3.58 | 11.9 | 3.05 | Gonad |
| 52 | 30/10/2013 | Yotsukura Coast | 964 | 0.09 | ND | 3.51 | 7.4 | 3.37 | Gonad |
| 53 | 30/10/2013 | Yotsukura Coast | 964 | 0.084 | ND | 3.66 | ND | 3.97 | Gonad |
| 54 | 31/10/2013 | Ena rocky Coast | 965 | 0.08 | ND | 5.83 | 13.3 | 4.39 | Gonad |
| 55 | 31/10/2013 | Ena rocky Coast | 965 | 0.096 | ND | 3.33 | 8.14 | 4.08 | Gonad |
| 56 | 16/1/2014 | Yotsukura Coast | 1042 | 0.095 | ND | 3.81 | 13.9 | 3.72 | Whole Body |
| 57 | 16/1/2014 | Yotsukura | 1042 | 0.102 | 4.13 | 3.11 | 17.5 | 3.24 | Whole Body |
| 58 | 21/1/2014 | Ena rocky Coast | 1047 | 0.101 | 10.4 | 3.86 | 17.2 | 3.94 | Whole Body |
| 59 | 21/1/2014 | Ena rocky Coast | 1047 | 0.092 | 10.8 | 3 | 24.8 | 3.38 | Whole Body |
| 60 | 21/1/2014 | Ena rocky Coast | 1047 | 0.105 | 10.4 | 2.79 | 22.8 | 3.53 | Whole Body |
| 61 | 21/1/2014 | Ena rocky Coast | 1047 | 0.103 | ND | 2.93 | 6.67 | 3.07 | Gonad |
| 62 | 2/2/2014 | Yotsukura offshore | 1059 | 0.039 | 44.5 | 8.01 | 135 | 6.59 | Gonad |
| 63 | 2/2/2014 | Ena rocky offshore | 1059 | 0.08 | 15.3 | 3.91 | 35.8 | 4.35 | Gonad |
| 64 | 2/2/2014 | Ena rocky offshore | 1059 | 0.077 | 18.9 | 3.7 | 46.3 | 3.84 | Gonad |
| 65 | 26/5/2014 | Ena rocky offshore | 1172 | 0.111 | 10.7 | 3.11 | 28.7 | 4.12 | Gonad |
| 66 | 26/5/2014 | Ena rocky offshore | 1172 | 0.046 | 9.73 | 3.98 | 26.5 | 4.85 | Gonad |
| 67 | 27/5/2014 | Yotsukura Coast | 1173 | 0.111 | ND | 3.63 | 7.94 | 3.21 | Whole Body |
| 68 | 27/5/2014 | Yotsukura Coast | 1173 | 0.108 | 4.26 | 2.9 | 5.06 | 3.17 | Whole Body |
| 69 | 27/5/2014 | Yotsukura Coast | 1173 | 0.118 | 3.47 | 3.03 | 7.18 | 3.31 | Whole Body |
| 70 | 27/5/2014 | Yotsukura Coast | 1173 | 0.107 | ND | 3.48 | 3.26 | 3.11 | Gonad |
| 71 | 27/5/2014 | Yotsukura Coast | 1173 | 0.104 | ND | 3.07 | 9.56 | 3.04 | Gonad |
| 72 | 28/5/2014 | Yotsukura offshore | 1174 | 0.082 | 11.3 | 3.93 | 28.4 | 4.29 | Gonad |
| 73 | 28/5/2014 | Yotsukura offshore | 1174 | 0.096 | 20.1 | 3.61 | 50 | 3.87 | Gonad |
| 74 | 29/5/2014 | Ena rocky Coast | 1175 | 0.112 | 3.84 | 3.27 | 12.6 | 3.43 | Whole Body |
| 75 | 29/5/2014 | Ena rocky Coast | 1175 | 0.113 | ND | 2.85 | 9.3 | 3.33 | Whole Body |
| 76 | 29/5/2014 | Ena rocky Coast | 1175 | 0.111 | ND | 3.85 | 11.5 | 3.18 | Gonad |
| 77 | 29/5/2014 | Ena rocky Coast | 1175 | 0.11 | ND | 2.93 | 5.73 | 3.59 | Whole Body |
| 78 | 29/5/2014 | Ena rocky Coast | 1175 | 0.113 | ND | 3.22 | 3.55 | 2.95 | Gonad |
| 79 | 29/5/2014 | Ena rocky Coast | 1175 | 0.106 | ND | 3.26 | ND | 3.32 | Gonad |
| 80 | 29/5/2014 | Ena rocky Coast | 1175 | 0.105 | ND | 3.33 | ND | 3.23 | Gonad |
| 81^*^ | 27/8/2018 | Yotsukura Coast | 2726 | 0.005 | ND | 0.021 | 0.13 | 0.004 | Gonad |
| 82 | 27/8/2018 | Yotsukura Coast | 2726 | 0.092 | ND | 0.27 | ND | 0.26 | Gonad |
| 83 | 27/8/2018 | Yotsukura Coast | 2726 | 0.092 | ND | 0.24 | ND | 0.26 | Gonad |
| 84 | 27/8/2018 | Yotsukura Coast | 2726 | 0.092 | ND | 0.27 | ND | 0.25 | Gonad |
| 85^*^ | 27/08/2018 | Yotsukura Coast | 2726 | 0.005 | ND | 0.026 | 0.14 | 0.006 | Gonad |
| 86^*^ | 27/08/2018 | Yotsukura Coast | 2726 | 0.005 | ND | 0.019 | 0.17 | 0.005 | Gonad |

^*^Radiocesium measurement based on the dry weight (DW) of sample.
