# Supplementary material for: Contamination of sea urchin Mesocentrotus nudus by radiocesium released during the Fukushima Daiichi Nuclear Power Plant accident
Source: PLoS One. 2022 Aug 15;17(8):e0269947. doi: 10.1371/journal.pone.0269947 (PMC9377606; doi:10.1371/journal.pone.0269947)
Supplement: S3 Table — AIC = −220.05. (DOCX) [file pone.0269947.s003.docx]

**S3 Table. Results from GLM analysis of the ^137^Cs counts reduction rate (*D*) per day in sea urchins, and a maximum likelihood test against the null model for the specific growth rate of the sea urchins.** AIC = −220.05

| **Model selected** | **ΔAIC^a^** | **χ^2^** | ***P*-value** |
| --- | --- | --- | --- |
| Specific growth rate (SGR) of sea urchin | 1.85 | 8.72 | <0.05 |

^a^Difference between the best model and the null model AIC.
