# Supplementary material for: Contamination of sea urchin Mesocentrotus nudus by radiocesium released during the Fukushima Daiichi Nuclear Power Plant accident
Source: PLoS One. 2022 Aug 15;17(8):e0269947. doi: 10.1371/journal.pone.0269947 (PMC9377606; doi:10.1371/journal.pone.0269947)
Supplement: S4 Table — (DOCX) [file pone.0269947.s004.docx]

**S4 Table. Summary statistics for the regression slopes of the ^137^Cs radioactivity (total number of ^137^Cs concentration), effective half-life (*T*_eff_, day), and ecological half-life (*T*_eco_, day) in seawater from the Yotsukura and Ena stations.**

| **Study area** | **Coefficient of determination**  **(*R^2^*)** | ***p*-value** | **Coefficient^*^** | | **Effective half-life**  **(*T*_ₑff_) days** | **Ecological half-life**  **(*T*_eco_) days** |
| --- | --- | --- | --- | --- | --- | --- |
|  |  |  | ***C*_0_ (Bq/L)** | ***λ*_eco_ (d^-1^)** |  |  |
| **Yotsukura station** | 0.11 | <0.05 | 0.091 | -0.0011 | 642 | 681 |
| **Ena**  **station** | 0.23 | <0.05 | 0.083 | -0.0015 | 466 | 487 |

^*^Coefficient for the following exponential equation for the measured data: *C*_t_ = *C*_0_e^(-^*^λ^*^t)^. *C*_t_ is the ^137^Cs concentration at elapsed day *t*, *C*_0_ is the ^137^Cs concentration for the first measurement time, and *λ*_eco_ is the decay rate.
