# Supplementary material for: Contamination of sea urchin Mesocentrotus nudus by radiocesium released during the Fukushima Daiichi Nuclear Power Plant accident
Source: PLoS One. 2022 Aug 15;17(8):e0269947. doi: 10.1371/journal.pone.0269947 (PMC9377606; doi:10.1371/journal.pone.0269947)
Supplement: S5 Table — (DOCX) [file pone.0269947.s005.docx]

**S5 Table. Analysis of covariance (ANCOVA) for the relative value of ^137^Cs activity (natural log scale) changes which were used to calculate half-life (*T*_bio_ and *T*_eco_) in sea urchin.**

| **Source of variation** | **DF** | **Sum of Squares** | ***F* Ratio** | ***P*-value** |
| --- | --- | --- | --- | --- |
| Place (Lab expt., Yotsukura and Ena coast) | 2 | 10.78 | 132.04 | <0.0001 |
| Passing days after the FDNPP accident | 1 | 3.63 | 89.03 | <0.0001 |
