# Supplementary material for: Contamination of sea urchin Mesocentrotus nudus by radiocesium released during the Fukushima Daiichi Nuclear Power Plant accident
Source: PLoS One. 2022 Aug 15;17(8):e0269947. doi: 10.1371/journal.pone.0269947 (PMC9377606; doi:10.1371/journal.pone.0269947)
Supplement: S1 Fig — (DOCX) [file pone.0269947.s006.docx]

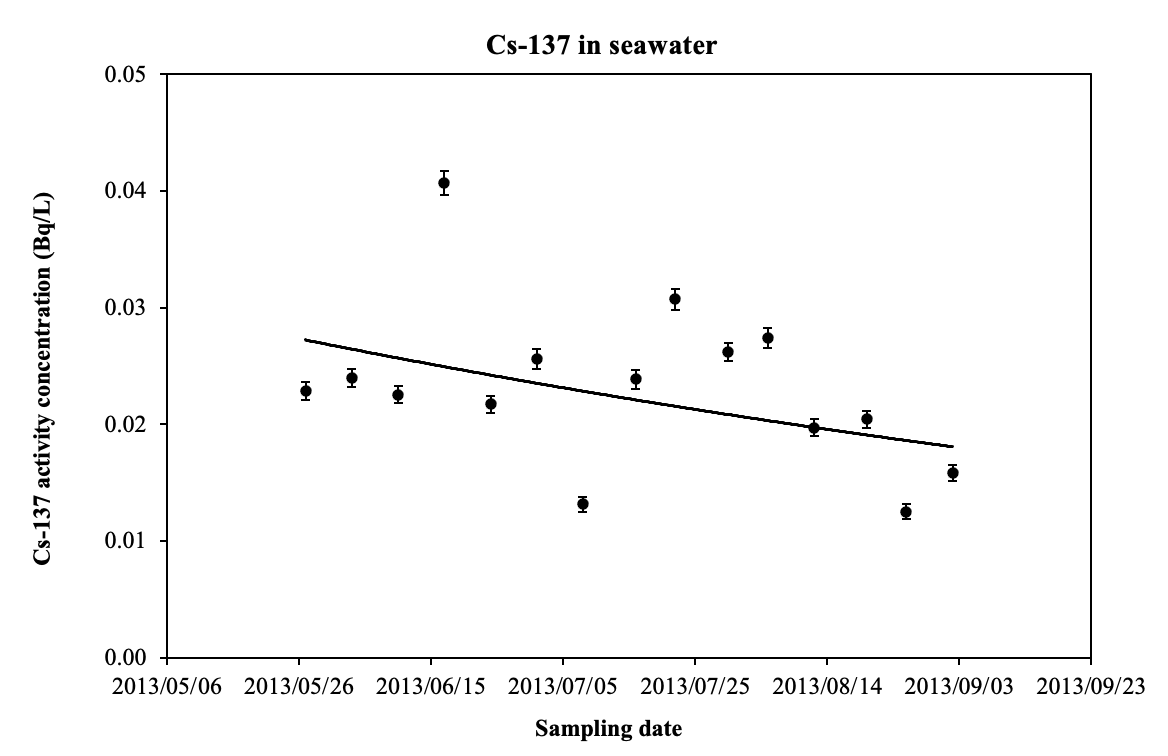

**S1 Fig. Trend of ^137^Cs activity concentration (Bq/L; Mean±SD) in seawater during the rearing experiment of sea urchin** **(Kaeriyama, 2017).**
