# Supplementary material for: Contamination of sea urchin Mesocentrotus nudus by radiocesium released during the Fukushima Daiichi Nuclear Power Plant accident
Source: PLoS One. 2022 Aug 15;17(8):e0269947. doi: 10.1371/journal.pone.0269947 (PMC9377606; doi:10.1371/journal.pone.0269947)
Supplement: S2 Fig — Solid and dashed lines represent statistically significant and insignificant regression slopes, respectively. (DOCX) [file pone.0269947.s007.docx]

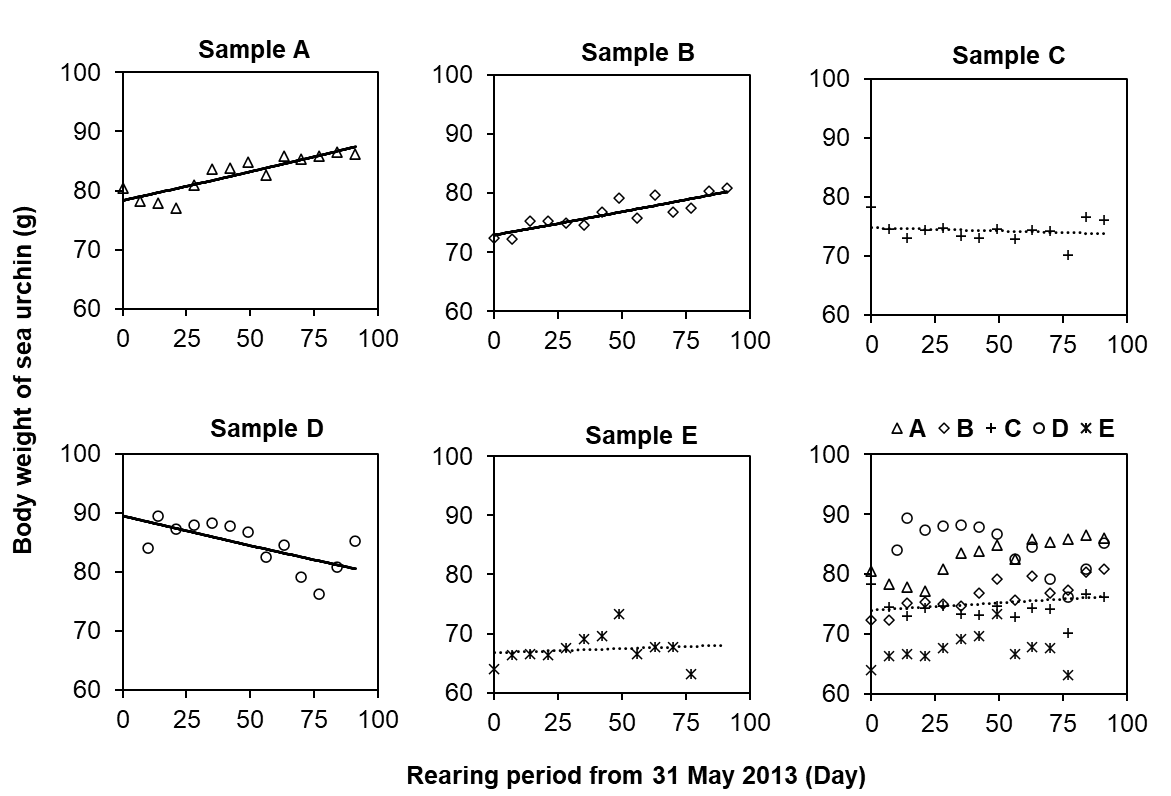


**S2 Fig. Temporal changes in the body weights of sea urchins during the rearing period (31 May–30 August 2013) in the laboratory.** Solid and dashed lines represent statistically significant and insignificant regression slopes, respectively.
