# Supplementary material for: Contamination of sea urchin Mesocentrotus nudus by radiocesium released during the Fukushima Daiichi Nuclear Power Plant accident
Source: PLoS One. 2022 Aug 15;17(8):e0269947. doi: 10.1371/journal.pone.0269947 (PMC9377606; doi:10.1371/journal.pone.0269947)
Supplement: S3 Fig — Filled and empty squares in the water data show our survey values and the NRA data, respectively. Solid lines indicate fitted exponential functions for 137Cs concentrations in sea urchins, seawater, marine plants, and sediment. Data below the detection limit were excluded. (DOCX) [file pone.0269947.s008.docx]

**S3 Fig. Spatial and temporal changes in the ^137^Cs activity concentrations in sea urchin gonads (Bq/kg-WW), seawater (Bq/L), marine plants (Bq/kg-WW), and sediment (Bq/kg-DW) collected from two fishing areas of the Fukushima Prefecture after the FDNPP accident.** Filled and empty squares in the water data show our survey value and the NRA data, respectively. Solid lines indicate fitted exponential functions for ^137^Cs concentrations in sea urchins, seawater, marine plants, and sediment. Data below the detection limit were excluded.
