# Supplementary material for: Contamination of sea urchin Mesocentrotus nudus by radiocesium released during the Fukushima Daiichi Nuclear Power Plant accident
Source: PLoS One. 2022 Aug 15;17(8):e0269947. doi: 10.1371/journal.pone.0269947 (PMC9377606; doi:10.1371/journal.pone.0269947)
Supplement: S4 Fig — (DOCX) [file pone.0269947.s009.docx]

 **S4 Fig. Uptake of ^137^Cs through food in sea urchins (Bq/Kg-WW) collected from the Yotsukura fishing ground in the Fukushima Prefecture after the FDNPP accident.**
